# A Retrospective Review Following the Addition of Clonidine to a Neonatal Abstinence Syndrome Treatment Algorithm

**DOI:** 10.3389/fped.2021.632836

**Published:** 2021-06-07

**Authors:** Mohammad Y. Bader, Nahla Zaghloul, Ashley Repholz, Nadia Nagy, Mohamed N. Ahmed, Leslie Thompson, Ranjit I. Kylat

**Affiliations:** ^1^Department of Pediatrics, University of Arizona Health Sciences, Tucson, AZ, United States; ^2^Banner University Medical Center, Tucson, AZ, United States; ^3^Las Vegas Children's Hospital, Las Vegas, NV, United States

**Keywords:** newborn, substance withdrawal syndrome, morphine, clonidine, neonatal opioid withdrawal syndrome, neonatal abstinence syndrome, withdrawal symptoms

## Abstract

**Objective:** To investigate the outcomes associated with the implementation of a neonatal abstinence syndrome (NAS) treatment algorithm utilizing dual therapy with morphine sulfate and clonidine in a level four neonatal intensive care unit (NICU).

**Study Design:** A cohort of neonates (≥35 weeks gestation) born at an academic tertiary medical center between January 1, 2015 and December 31, 2018 who were diagnosed with NAS were retrospectively evaluated following the implementation of a new NAS treatment algorithm. Neonates were categorized in two groups based on if they were treated pre- or post-implementation of the protocol. The primary efficacy outcome was length of hospital stay. Secondary outcomes included the incidence of adverse drug reactions, length of treatment for NAS, and maximum as well as total cumulative dose of each medication used to treat NAS.

**Results:** The implementation of this NAS treatment algorithm significantly reduced the length of hospital stay (30 days vs. 20 days, *p* = 0.001). In addition, there was a significant decrease in duration of morphine sulfate exposure as well as cumulative dose of morphine required to successfully treat a neonate with NAS in the post-implementation group (26 days vs. 15 days, *p* = 0.002 and 6.9 mg/kg vs. 3.4 mg/kg, *p* = 0.031).

**Conclusion:** Addition of clonidine to morphine sulfate as initial therapy for NAS significantly reduced the cumulative exposure as well as duration of exposure to morphine sulfate compared to morphine monotherapy and decrease length of hospital stay.

## Introduction

Neonatal abstinence syndrome (NAS) is described as a constellation of symptoms resulting from alterations in the central and autonomic nervous systems secondary to abrupt cessation of fetal exposure to substances taken by the mother during pregnancy (1, 2). These substances include illicit drugs such as heroin, prescription pain medications, and methadone prescribed for treating chronic opioid use. NAS can cause severe withdrawal symptoms requiring prolonged treatment. Prolonged length of hospital stays can also burden institutions financially as increasing studies have shown an increase in cost of neonatal intensive unit (NICU) care associated with NAS (1–4).

The number of neonates diagnosed with NAS has nearly quadrupled from 2004 to 2013, during which time the median length of hospital stay increased from 13 to 19 days (4–6). Hospitalization of NAS neonates is required for the treatment of withdrawal symptoms. These are managed by non-pharmacologic therapy when mild and with pharmacologic therapy when supportive treatment fails to control symptoms, typically with morphine sulfate as the primary agent (1, 3). Morphine monotherapy has been used as first line therapy and has been shown to ameliorate the severity of withdrawal symptoms, and is associated with prolonged length of hospital stay (1).

While opioids tend to be the most common culprit of NAS given the current opioid epidemic as well as the promotion of methadone treatment programs; poly-drug exposure is not uncommon and thus provokes the question of the most appropriate drug of choice for the treatment of these neonates (3, 7, 8). Phenobarbital, methadone, diluted tincture of opium, clonidine and buprenorphine have all been shown to have a role in the treatment of NAS (1–5, 9, 10).

Phenobarbital (PB), a ⋎-amino butyric acid agonist, while easy to administer with once daily dosing, has the potential to over sedate, increase overall duration of therapy, and potentially cause neuro-cognitive impairment (9–13). Alternatively, clonidine, an alpha_2_-adrenergic agonist, has improved safety profile compared to phenobarbital and has some neuroprotective effects while decreasing NAS symptoms by reducing the sympathetic outflow (14, 15). In a prospective study comparing clonidine to PB as an adjunct medication to treat NAS, Surran et al. found that in both adjunct therapy groups the length of hospital stay was shortened compared to receiving morphine alone (16). The study noted that PB as an adjunct to therapy had a shorter length of hospitalization, which was not significant when compared to clonidine adjunct therapy but it exposed infants to more prolonged drug exposure since an outpatient dose tapering was necessary. PB has the potential to cause neuronal apoptosis which makes clonidine potentially more favorable to avoid the prolonged exposure to PB (17).

Despite potential advantages of clonidine based upon animal models, there has not been a demonstration of differential safety in humans. This study is aimed at assessing the change in length of hospital stay after the implementation of a new morphine and clonidine dual therapy protocol compared to morphine monotherapy.

## Methods

### Study Population

This study is a single-center, retrospective study of neonates who were admitted to the NICU at an academic medical center and subsequently diagnosed with NAS between January 1, 2015 and December 31, 2018. This study was approved by the Institutional Review Board of Banner University Medical Center, Tucson. Neonates were categorized into groups based on treatment. Treatment groups were divided into two the first, group 1, which was prior to implementation of the new neonatal treatment algorithm protocol, was treated only with morphine. Group 2, after the treatment protocol was introduced received both morphine and clonidine. The new treatment algorithm was implemented August 1, 2016, therefore the pre-implementation group consisted of patients admitted from January 1, 2015 through July 31, 2016 and the post-implementation group consisted of those admitted August 1, 2016 through December 31, 2018. There was no patient overlap between the two treatment groups. The cohort was identified utilizing ICD-10 codes 96.1 and 96.2 which code for NAS or neonatal withdrawal syndrome.

**Figure 1:**
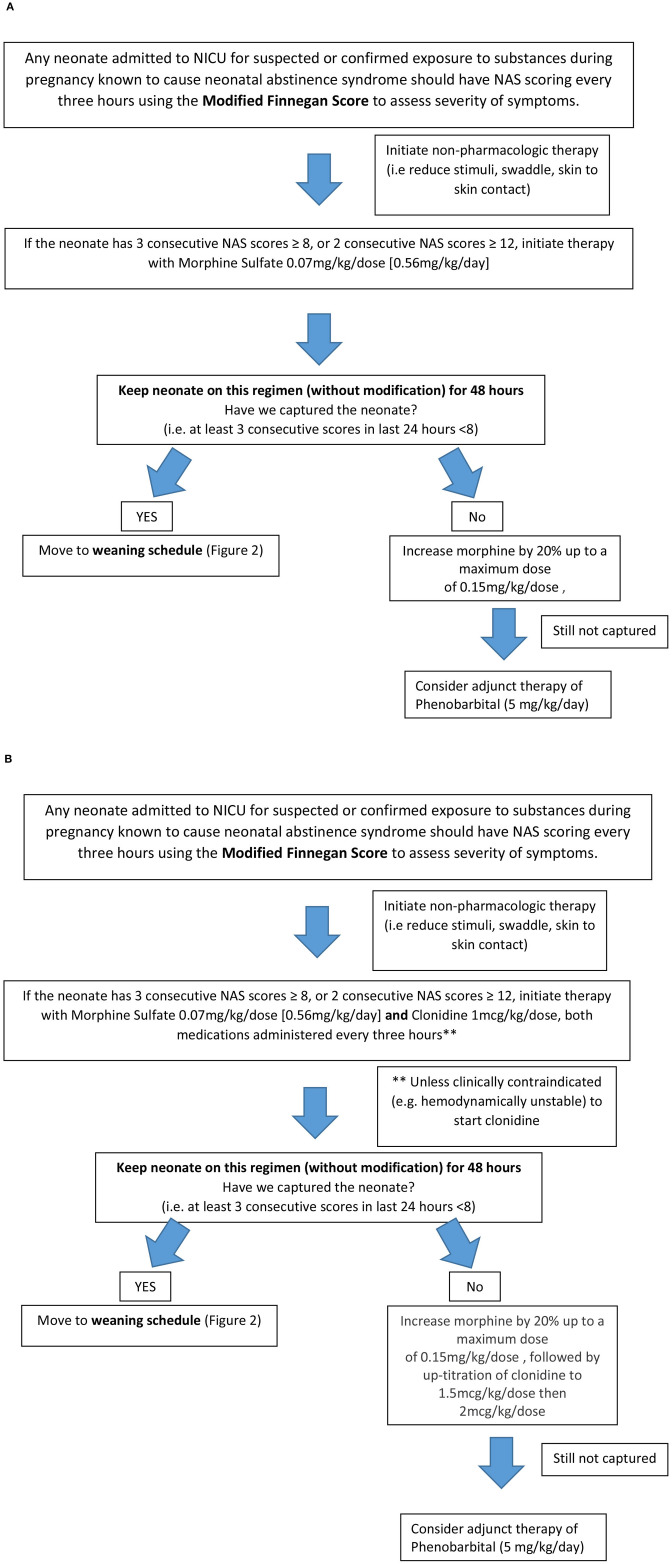
Neonatal Abstinence Syndrome Treatment Algorithm. **(A)** Group 1 pre-implementation Neonatal Abstinence Syndrome Treatment Algorithm (2/2014). **(B)** Group 2 Post-implementation BUMC-T Neonatal Abstinence Syndrome Treatment Algorithm (8/2016).

**Figure 2:**
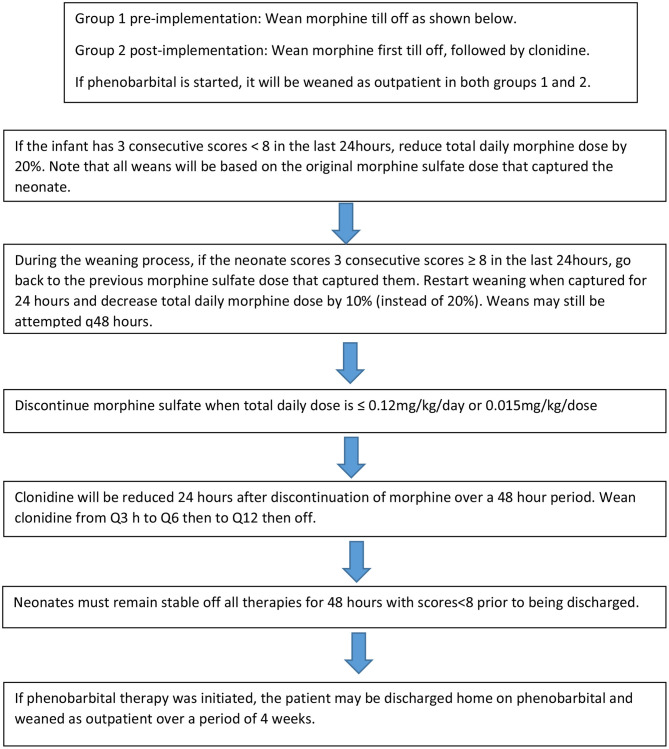
Neonatal Abstinence Syndrome Treatment Algorithm Weaning Schedule.

Patients from this cohort were included if they were admitted and discharged from the NICU within the study time period, were >35 weeks gestation, and had been maintained on room air following admission. Exclusion criteria included presence of congenital anomalies, diagnosis of fetal alcohol syndrome, inability to take enteral medications for >2 days, seizure disorder unrelated to NAS and hypoxic Ischemic Encephalopathy (HIE). The institution's previous standard of care for NAS prior to the implementation of the treatment algorithm (Figure 1A) was guided by Modified Finnegan Scores (MFS), which prompted initiation of morphine sulfate. Weaning of medications was also managed by the provider according to MFS (Figure 2). PB if initiated as adjunct was continued upon hospital discharge and managed by the patient's primary care provider on an outpatient basis.

The NAS treatment algorithm implemented in August 2016 utilized MFS to assess severity of symptoms and initiate treatment if the neonate had three consecutive scores greater than or equal to eight (Figure 1B). If this occurred, morphine sulfate oral solution in combination with clonidine oral solution (20 mcg/mL) were started at 0.07 mg/kg/dose and 1 mcg/kg/dose, respectively, every 3 h (q3h) to coincide with MFS. The neonate remained on this initial regimen for the first 48 h, at which point scores were reassessed to determine if the neonate was captured on these doses. Capture was defined as the presence of at least 3 consecutive MFS less than eight in the previous 24 h. If the neonate was captured, the weaning process began by reducing morphine sulfate by 20% of the original total daily morphine dose every 48 h until reaching 0.12 mg/kg/day or less, at which point it was discontinued (Figure 2). After morphine had been discontinued for 24 h, clonidine sulfate was tapered by decreasing the frequency on 3 consecutive days until discontinued. For example, if a neonate was captured on clonidine 4 mcg orally q3h, the first wean would transition from clonidine 4 mcg q3h to 4 mcg q6h on day 1, q6h to q12h on day 2, and discontinued on day 3. After all medications had been stopped for 48 h the patient was discharged home. If the neonate continued to illustrate significant signs of withdrawal as illustrated by high MFS despite maximum doses of morphine sulfate (1.2 mg/kg/day) and clonidine (16 mcg/kg/day), or the patient experienced seizures associated with withdrawal, phenobarbital (5 mg/kg/day) therapy was added. Then, the patient was discharged home on phenobarbital to be weaned outpatient.

The primary outcome of this study was length of hospital stay. Secondary outcomes included length of treatment for NAS and the cumulative doses of medications used to treat NAS. Safety of the treatment algorithm was assessed utilizing the Naranjo Adverse Drug Reaction Probability Scale, which describes the probability that the adverse drug reaction seen was caused by the drug in question (18).

Statistical analysis was performed using SAS software^®^ (version 9.4, SAS Institute Inc., Cary, NC, USA). Continuous variables were analyzed using two -sided student *t*-test with a predefined significance set at *p* < 0.05. Mann- Whitney U test was utilized with continuous variable that follow non-normal distributions. X^2^ or Fisher's Exact Test were used for categorical variables.

## Results

**Figure 3:**
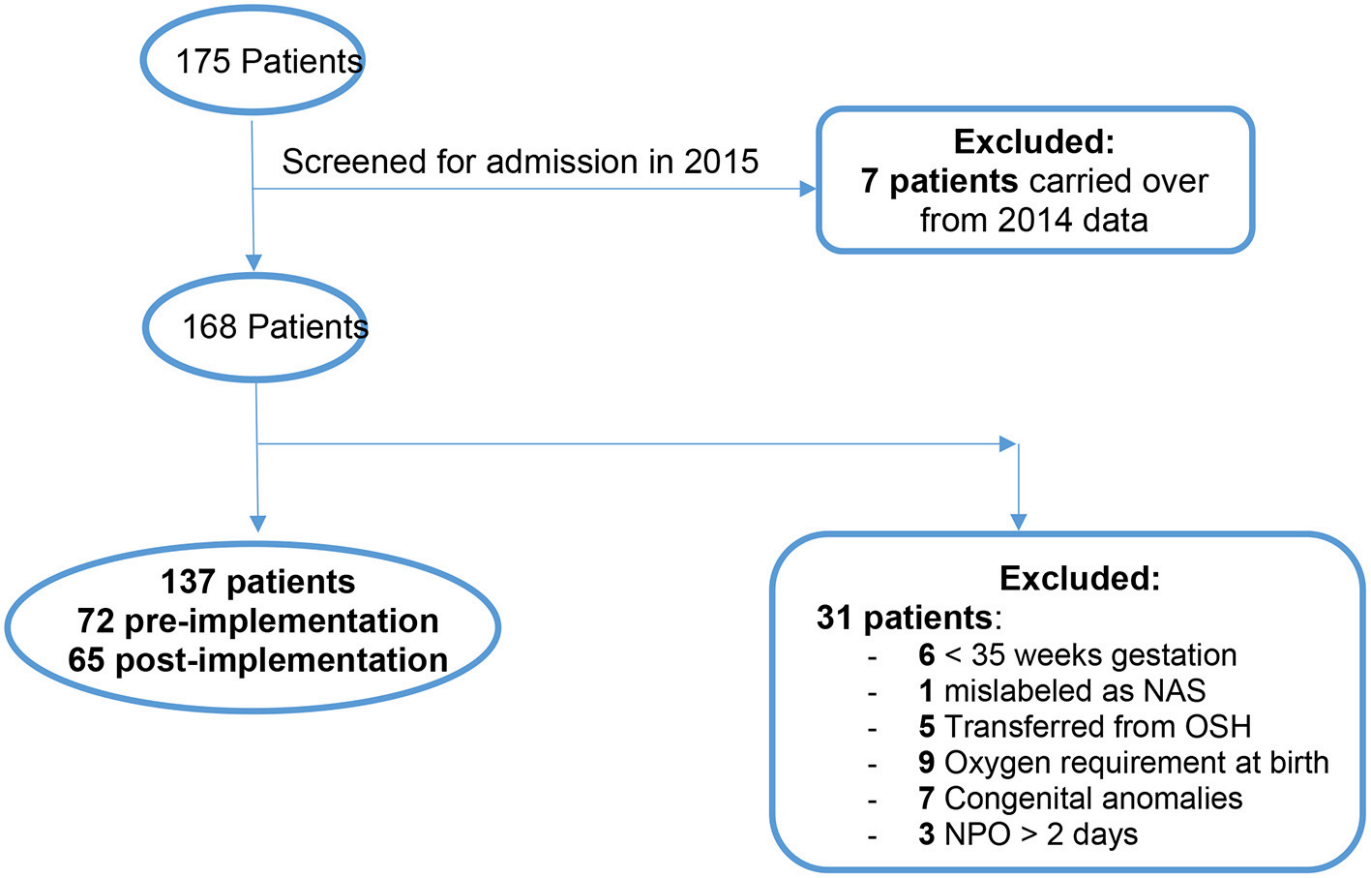
Flow diagram of the study population.

There were 175 total patients identified with the ICD-10 codes related to NAS within our time parameters (Figure 3). Of those, 7 patients were excluded from the study because they were admitted in 2014 and later discharged in January 2015. One hundred and sixty eight patients were eligible to screen for inclusion in the study. After screening was completed with the mentioned exclusion criteria there were a total of 137 neonates remaining for analysis.

When comparing patient demographic data between the treatment groups, there was no statistically significant difference in the sex, race, birth weight, number of small for gestational age (SGA) neonates, head circumference at birth, maternal methadone use, maternal use of psychotropic medications, or maternal smoking (Table 1). The groups were predominantly a white, non-Hispanic population with a male to female ratio in the pre- to post-implementation group of 2:1, respectively, difference was not significant. The groups were predominately not SGA neonates with the exception of 7 in the pre- implementation group and 4 in the post-implementation group. Both groups had similar maternal methadone use *in-utero*, and about one third of the neonates in each group were exposed to psychotropic medications.

**Table 1:** Demographics of participants in both study groups.

	**Pre-implementation Group I (morphine) (*N* = 72)**	**Post-implementation Group II (morphine and clonidine) (*N* = 65)**	***P*-value**
**Sex, no. (%)**			
Female	25 (35%)	31 (47%)	0.37
Male	47 (65%)	34 (53%)	
**Race, no. (%)**			
White, non-Hispanic	55 (76%)	51 (78%)	0.90
Other	17 (24%)	14 (21%)	
Birth weight (Kg)^*^	2.92 ± 0.7	3.02 ± 0.6	0.17
Small for gestational age, no. (%)	7 (10%)	4 (6%)	0.68
Head circumference at birth (cm)^*^	32.9 ± 2.1	32.7 ± 2.2	0.25
Maternal methadone, no. (%)	40 (56%)	39 (60%)	0.38
Maternal psychotropic medications	24 (33%)	23(35%)	0.90
Maternal smoking	34 (47%)	33 (50%)	0.69

* *Mean ± SD*.

Neonatal urine and meconium toxicology profiles were assessed to determine exposures between groups. There were no significant differences in each substance analyzed in both the meconium and urine studies between the groups (Table 2). Neonates in this population are generally exposed to at least opiates, polysubstance exposure is less uncommon. Maternal urine toxicology, if reported in the neonate's chart, was also compared and found to be similar between groups (Table 3). There was no significant difference in severity of withdrawal between pre and post implementation groups as assessed by Modified Finnegan Scores in the first 96 h of life.

**Table 2:** Neonatal urine and meconium toxicology profiles.

	**Pre-implementation Group I (morphine) (*N* = 72)**	**Post-implementation Group II (morphine and clonidine) (*N* = 65)**	***P*-value**
**Neonate meconium, no. (%)**			
Opiates	29 (40)	37 (57)	0.729
Cocaine	5 (7%)	4 (6%)	>0.999
Amphetamine	11 (15%)	30 (46%)	0.352
Cannabinoids	12 (17%)	10 (16%)	>0.999
Methadone	28 (38%)	20 (31%)	0.71
Phyencyclidine	–	–	–
Not collected	18 (25%)	14 (22%)	0.699
**Neonate urine, no. (%)**			
Amphetamines	11 (15%)	30 (46%)	0.299
Barbiturates	12 (17%)	8 (12%)	0.689
Benzodiazepines	–	–	–
Benzoylecgonine	–	–	–
Delta 9 THC	12 (17%)	10 (16%)	>0.999
Opiates	29 (40%)	37 (57%)	0.415
Not collected	7 (10%)	6 (9%)	>0.999

**Table 3:** Maternal urine toxicology profile.

	**Pre-implementation Group I (morphine) (*N* = 72)**	**Post-implementation Group II (morphine and clonidine) (*N* = 65)**	***P*-value**
**Maternal urine, no. (%)**			
Amphetamines	9 (13%)	25(38%)	0.357
Barbiturates	8 (11%)	6 (9%)	>0.999
Benzodiazepines	–	–	–
Benzoylecgonine	–	–	–
Delta 9 THC	7 (10%)	6 (9%)	>0.999
Opiates	21 (29%)	28 (43%)	0.467
Not available	33 (46%)	29 (45%)	>0.999

The primary outcome of length of hospital stay was significantly decreased from 30 days pre-implantation to 20 days post-implementation (*p* = 0.001) (Table 4). The duration of treatment was significantly reduced from a median of 26 days in the pre-implementation to 15 days in the post-implementation group (*p* = 0.002). The cumulative dose of morphine sulfate exposure was also significantly cut in half from 6.9 mg/kg pre-implementation to 3.4 mg/kg post-implementation (*p* = 0.031). There were no documented adverse effects such as arrhythmias, changes in heart rate or blood pressure, or deaths due to NAS in either the pre- or post-implementation groups. Heart rate decreased by an average of 8 bpm in infants receiving clonidine at 1 mcg/kg/dose. In infants receiving clonidine, there was no change in blood pressure and no cases of rebound hypertension likely due to the slow wean of clonidine over a period of 3 days.

**Table 4:** Neonatal outcomes pre and post implementation of NAS treatment algorithm.

	**Pre-implementation Group I (morphine) (*N* = 72)**	**Post-implementation Group II (morphine and clonidine) (*N* = 65)**	***P*-value**
Hospital length of stay (Days)	30	20	0.001^*^
Length of NAS treatment	28	18	0.001^*^
Total morphine dose (mg)	6.9	3.4	0.031^*^
Patients discharged on phenobarbital	4 (5%)	2 (3%)	0.09
Breastfeeding at discharge, no. (%)	14 (19%)	17 (26%)	0.26
Any breastfeeding	27 (37%)	31 (47%)	0.15
Days on morphine	26.2	14.7	0.002^*^
Discharge with birth parents	24 (33%)	20 (31%)	0.85

* *Statistical significance. The bold values are meant for statistically significant values*.

## Discussion

This is a study comparing the efficacy and safety of the previous standard of care, morphine sulfate as monotherapy, to dual therapy of morphine sulfate and clonidine in the treatment of NAS. Similar to Raffaeli et al., we were able to identify a significant decrease in length of stay when adding clonidine as an adjunct to morphine monotherapy (13). Decreasing hospital stay not only decreases the total cost, but more importantly decreases neonatal exposure to treatment. This has been demonstrated by a decrease in the duration of exposure to morphine sulfate as well as cumulative morphine sulfate dose with dual therapy (Table 4). This remains an important clinical outcome as duration of opioid exposure *in utero* as well as postnatally are not benign exposures as described by Naranjo et al. (18). The dose and the duration of opiate exposures play a role in the size, weight and production of neurotransmitters—specifically within the GABA system—which interfere with normal neuronal transmission that may ultimately lead to inappropriate excitability and increased seizure potential (18). Given these findings, it is imperative that we minimize the exposure to opioids postnatally when possible.

Four patients in the pre-implementation group received phenobarbital and 2 patients in the post-implementation group received phenobarbital and they were all discharged home on phenobarbital. The average time of weaning off phenobarbital was 4 weeks.

Outpatient weaning of phenobarbital, usually dose was cut in half by 2 weeks and then discontinued by 4 weeks after discharge.

This was a retrospective study and as such there are limitations, including susceptibility to selection bias. It is a single center study and might not be representative of all substance exposures across the entire country.

A limitation to this study is the lack of assessment of adherence to the treatment protocol at the time of study, although assessments were made retrospectively. While treatment algorithms are a great way to promote unified care across providers, we realize that not every neonate will fit the algorithm. Neonatal practitioners were encouraged to follow the dose increase as well as weaning instructions as closely as possible with the idea that some children may be able to be weaned at a faster rate, and that the protocol should not slow this process down if appropriate. There was full adherence to the protocol in initiation and up-titration till capture of the infant. The non-adherence although rare, was in the percent of dose weaning since some babies had lower scores and could be weaned faster and others did not. Non-adherence rate was 12% in the pre-implementation group and 9% in the post-implementation group. Another limitation related to the implementation of a protocol is that a decrease in hospital length of stay could have been affected solely based on the implementation of a new NAS treatment algorithm. Although there was a protocol for morphine monotherapy with the addition of phenobarbital after reaching maximum morphine therapy, implementing a new standardized protocol limits the variability in treatment between NICU providers which could have affected the data.

Despite potential biases due to retrospective nature of the study, we attempted to control for nursing bias regarding the assessment of these neonates. While there may have been some inherent bias based on attention drawn to this specific population due to the implementation of the protocol, nurses were not re-educated on how to score neonates using the Modified Finnegan Score to avoid confounding the data. The scoring system is subjective in nature and has been shown to vary between nurses. The importance of routine education on scoring neonates remains an important part of the treatment of neonatal abstinence syndrome and therefore should not be ignored in future studies.

Our exclusion criterion were purposely stringent in order to capture neonates whose sole problem was NAS. While this provides the most accurate picture of the treatment of this population, some of the criterion may be reviewed for modification. These criterion primarily consist of oxygen requirements in the first 24 h of life and transition to the general pediatrics floor. An area where a vast majority of our patient population was excluded was in the transfer out of the NICU to the general pediatric ward due to the need to make room for neonates requiring acute care. While our hospitalists and pediatric residents were educated on the use of the protocol, it was found that these teams were much less comfortable with the use of the protocol and thus deviated more frequently, leading to longer lengths of stay compared to those neonates who finished treatment in the NICU. Further education for all medical staff on use of the treatment algorithm and Modified Finnegan Scoring or alternative scoring methods are needed for future treatment of these neonates on the pediatric floor (19).

The treatment of NAS has continued to change as the body of literature in this area expands. As the amount of evidence regarding negative impact of opioid exposure in this critical time of brain growth and development increases, the emphasis on non-pharmacologic treatments including swaddling, low-stimulus environments and an increased focus on mother-baby bonding and family-centered care where appropriate continues to surface as a potential primary therapy for neonates with NAS. This care is often optimized by use of single occupancy rooms with high family-centered therapy, which can be challenging for NICUs with a multiple-occupancy layout. Given the nature of this disease state, family and social issues are common in this patient population, further complicating the implementation of family-centered care.

Based on the results of this study we believe that combination therapy with morphine and clonidine is superior to morphine monotherapy, as it decreased length of stay and cumulative opioids given. Due to the potential harmful neurocognitive effects from further exposure to opiates and the financial consequences associated with increased length of NICU stays, it is crucial to continue to improve the treatment of NAS (20).

## Data Availability Statement

The original contributions presented in the study are included in the article/supplementary material, further inquiries can be directed to the corresponding author/s.

## Ethics Statement

The studies involving human participants were reviewed and approved by University of Arizona research and ethics board. Written informed consent from the participants' legal guardian/next of kin was not required to participate in this study in accordance with the national legislation and the institutional requirements.

## Author Contributions

All authors have made a substantial, direct and intellectual contribution to this work and approved it for publication. MYB, NZ, MNA, and RIK contributed to the conception, organization and oversight of the study, data analysis and interpretation and revision of the manuscript draft and final approval of the version to be published. AR, NN, and LT contributed to implementation of the study, data interpretation, writing the original draft of the manuscript and final approval of the version to be published.

## Conflict of Interest

The authors declare that the research was conducted in the absence of any commercial or financial relationships that could be construed as a potential conflict of interest.
